# Supplementary figures and images for: CRL4Cdt2 ubiquitin ligase regulates Dna2 and Rad16 (XPF) nucleases by targeting Pxd1 for degradation
Source: PLoS Genet. 2020 Jul 21;16(7):e1008933. doi: 10.1371/journal.pgen.1008933 (PMC7394458; doi:10.1371/journal.pgen.1008933)

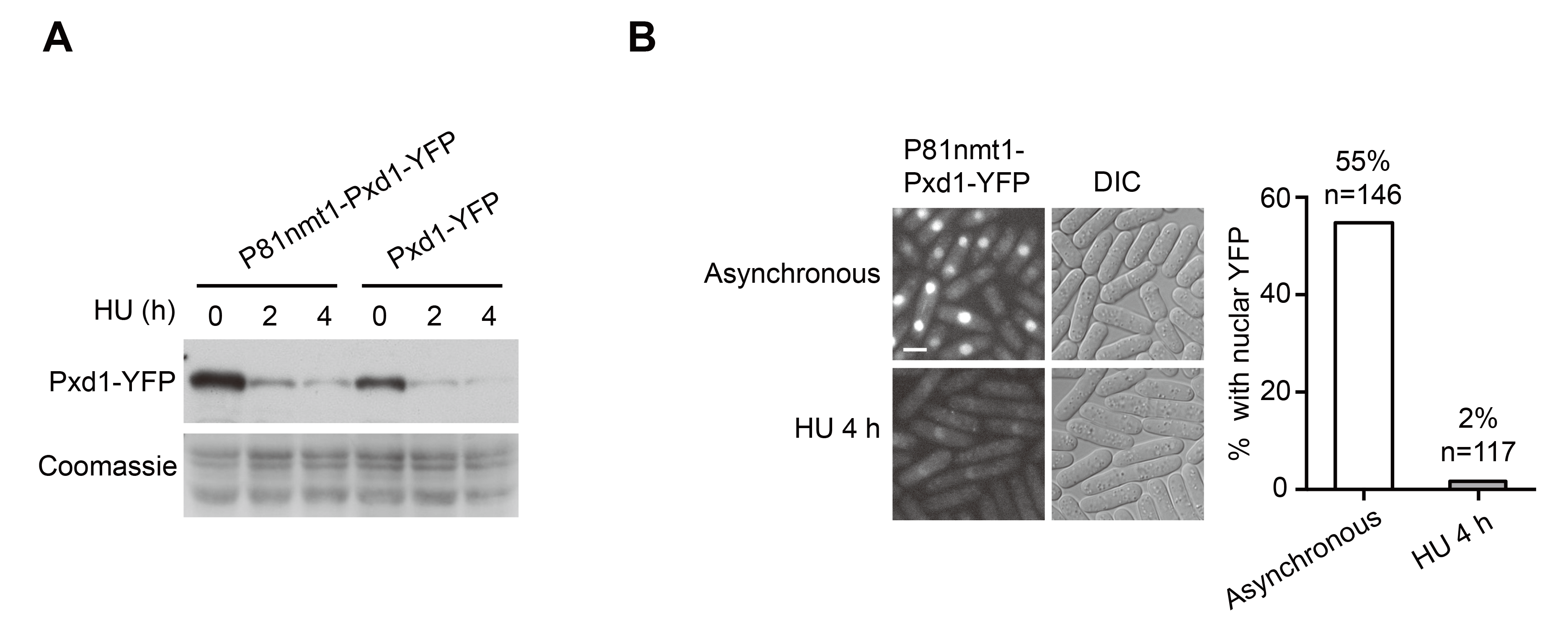

Supplement: S1 Fig — (A) The protein level of Pxd1-YFP expressed from the P81nmt1 promoter or its own promoter was examined in asynchronous cells and HU-treated cells. Cells were treated with 12 mM HU for 2 h or 4 h. (B) Micrographs (left) and quantitation (right) showing that HU treatment reduced the fluorescence signal of Pxd1-YFP expressed from the P81nmt1 promoter. Bar, 3 μm. n, the number of cells used for quantitation. (TIF) [file pgen.1008933.s001.tif]

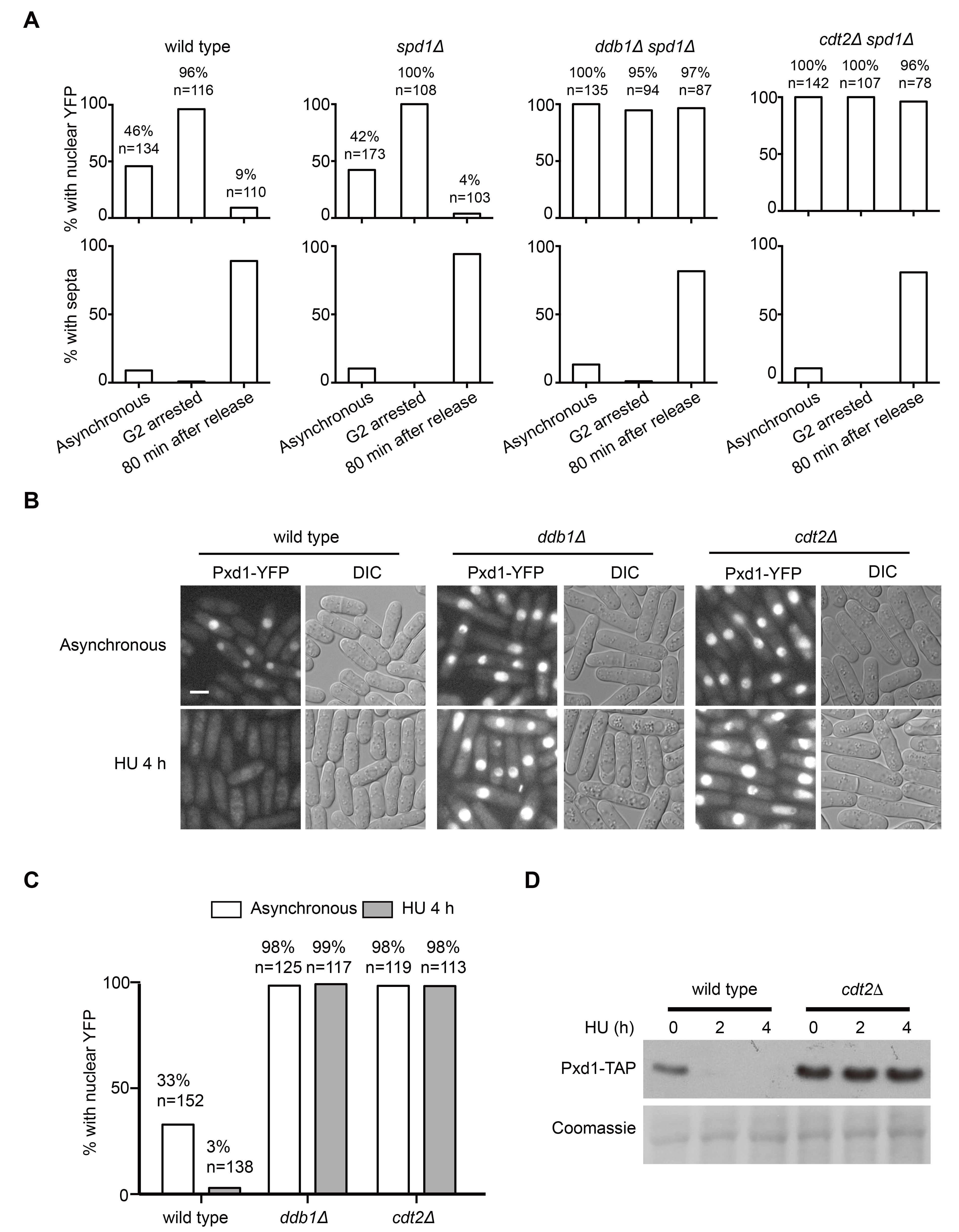

Supplement: S2 Fig — (A) Quantitation of the live cell imaging data shown in Fig 2B. The percentage of cells with nuclear YFP signal and the percentage of cells with septa are shown. n, the number of cells used for quantitation. (B-C) Micrographs (B) and quantitation (C) showing that HU treatment did not affect the fluorescence signal of Pxd1-YFP in ddb1Δ and cdt2Δ cells. Bar, 3 μm. (D) The protein level of Pxd1-TAP in asynchronous cells and HU-treated cells of wild type and the cdt2Δ mutant. (TIF) [file pgen.1008933.s002.tif]

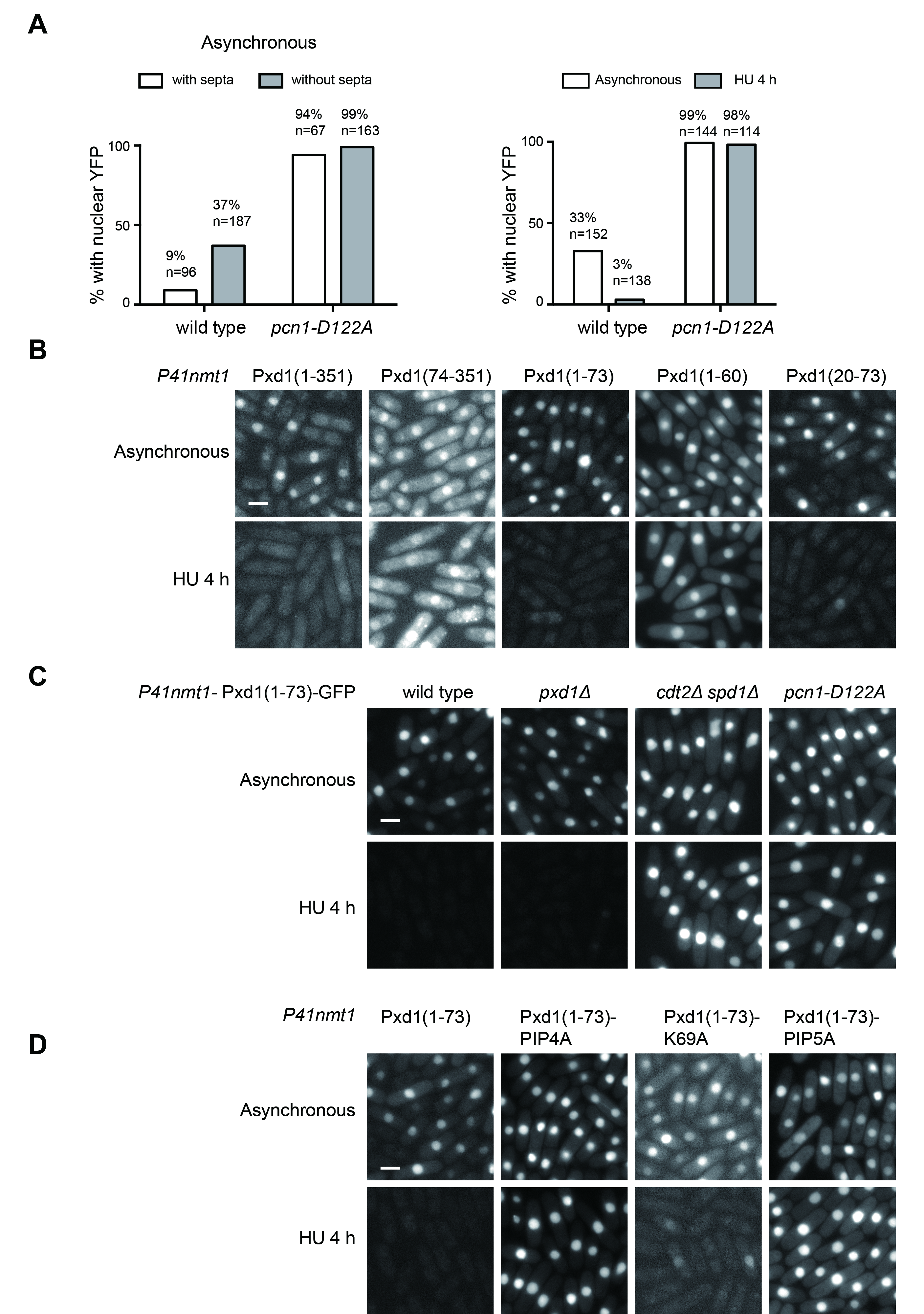

Supplement: S3 Fig — (A) Quantitation of the live cell imaging data shown in Fig 3A. The percentage of cells with nuclear YFP signal is shown. n, the number of cells used for quantitation. (B) The fluorescence signals of GFP-tagged full-length Pxd1 (351 amino acids) and truncated Pxd1 fragments. The expression was driven by the P41nmt1 promoter. The results are summarized in Fig 3C. (C) The fluorescence signal of Pxd1(1–73)-GFP in asynchronous and HU-treated cells of wild type, pxd1Δ, cdt2Δ spd1Δ, and pcn1-D122A mutants. The expression was driven by the P41nmt1 promoter. (D) The fluorescence signal of GFP-tagged Pxd1(1–73), Pxd1(1–73)-PIP4A, Pxd1(1–73)-K69A, and Pxd1-PIP5A in asynchronous and HU-treated cells. The expression was driven by the P41nmt1 promoter. Bars, 3 μm. (TIF) [file pgen.1008933.s003.tif]

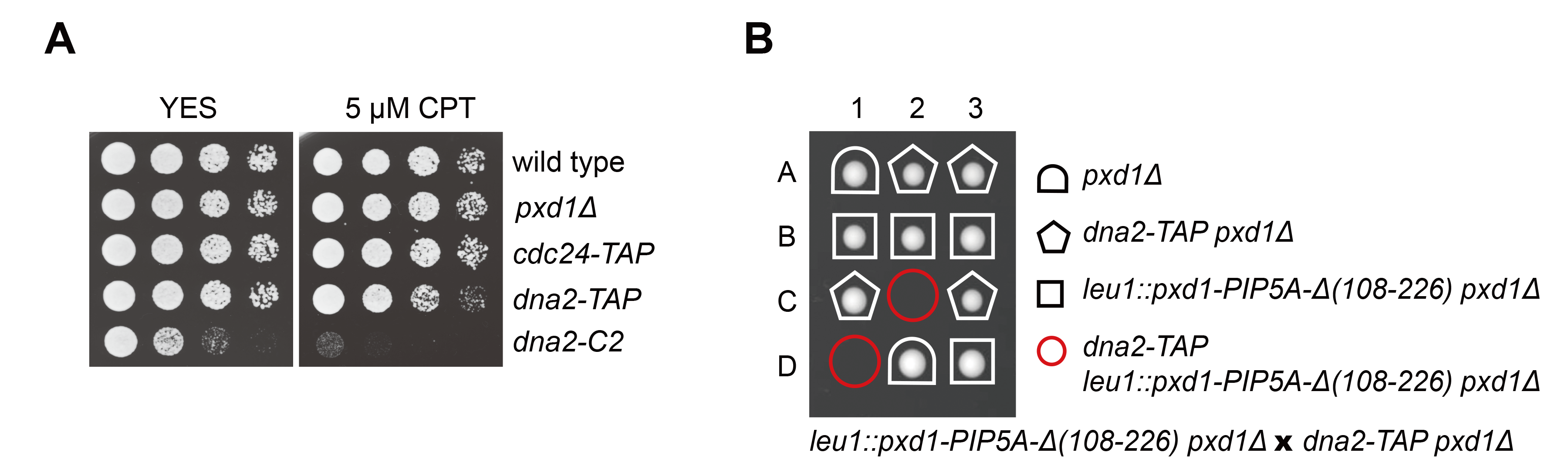

Supplement: S4 Fig — (A) dna2-C2 and dna2-TAP mutants exhibited sensitivity to CPT. Serial dilutions of strains were spotted on YES plates without and with CPT. (B) The synthetic lethality between pxd1-PIP5A and dna2-TAP was not rescued by the Pxd1 truncation mutation that abrogates Rad16 activation. (TIF) [file pgen.1008933.s004.tif]

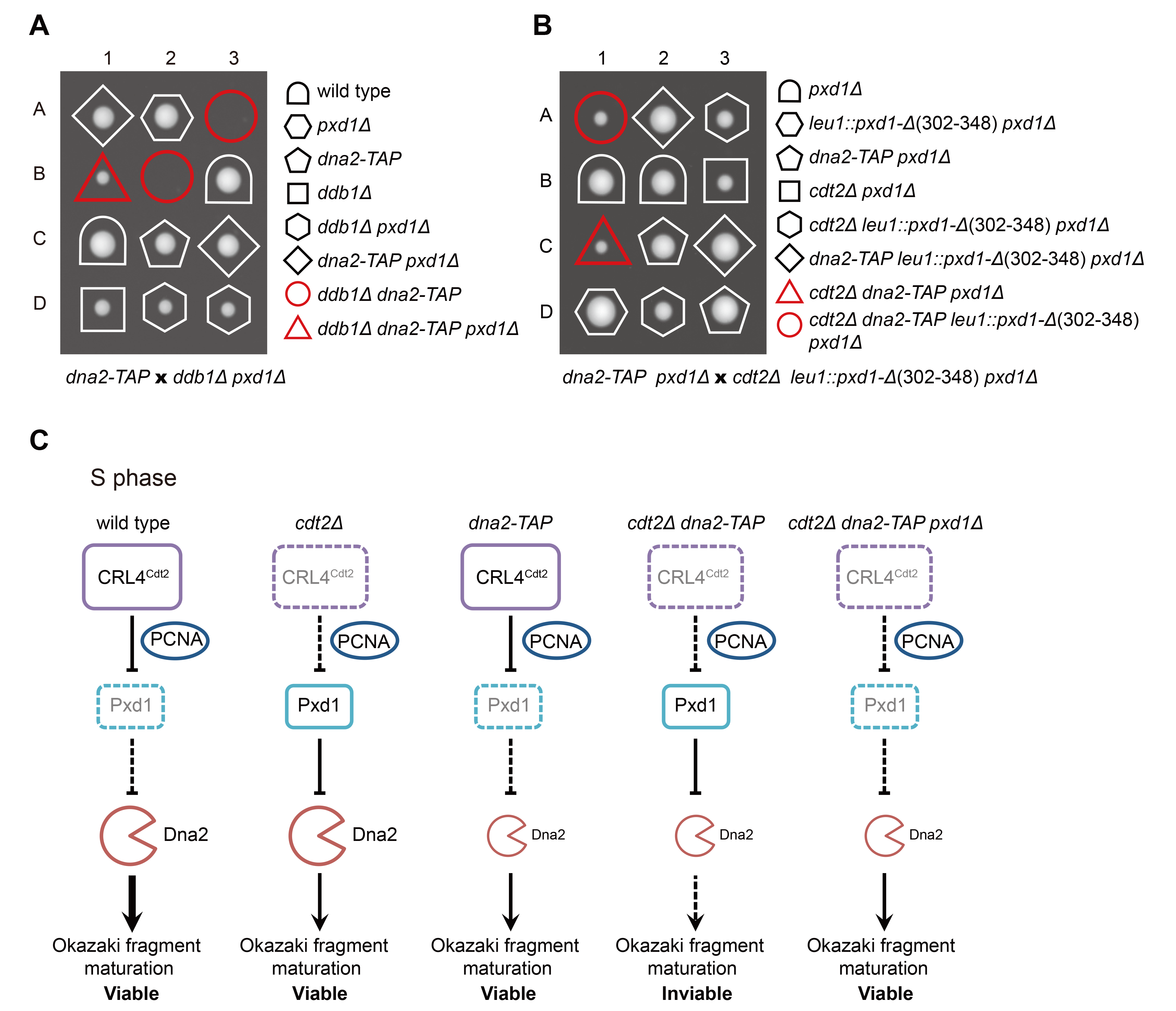

Supplement: S5 Fig — (A) ddb1Δ was synthetic lethal with dna2-TAP and this synthetic lethality was suppressed by the deletion of pxd1. (B) Introducing truncated Pxd1 with only Rad16-activation activity did not affect the growth of the cdt2Δ dna2-TAP pxd1Δ mutant. (C) Model explaining the synthetic lethality between cdt2Δ and dna2-TAP. (TIF) [file pgen.1008933.s005.tif]
