# Supplementary material for: CRL4Cdt2 ubiquitin ligase regulates Dna2 and Rad16 (XPF) nucleases by targeting Pxd1 for degradation
Source: PLoS Genet. 2020 Jul 21;16(7):e1008933. doi: 10.1371/journal.pgen.1008933 (PMC7394458; doi:10.1371/journal.pgen.1008933)
Supplement: S1 Table — (PDF) [file pgen.1008933.s006.pdf]

S1 Table. Fission yeast strains used in this study.

Strain	Mating type	Genotype	Fig.
DY6285	h+	*leu1-32::pJK148-pxd1-YFH(leu1) his3-D1 pxd1Δ::natMX*	1,2,3 S1
DY22859	h+	*leu1-32::pJK148-pxd1-YFH(leu1) his3-D1 pxd1Δ::natMX cdc25-22*	1
DY15007	h?	*leu1-32 his3-D1 pxd1-TAP::hphMX cdc25-22*	1
DY27193	h-	*leu1-32::pDual-P81nmt1-pxd1-YFH(leu1+) his3-D1*	S1
DY22864	h+	*leu1-32::pJK148-pxd1-YFH(leu1) his3-D1 pxd1Δ::natMX ddb1Δ::kanMX*	2
DY22868	h+	*leu1-32::pJK148-pxd1-YFH(leu1) his3-D1 pxd1Δ::natMX cdt2Δ::kanMX*	2
DY23101	h+	*leu1-32::pJK148-pxd1-YFH(leu1) his3-D1 pxd1Δ::natMX spd1Δ::hphMX cdc25-22*	2
DY23097	h+	*leu1-32::pJK148-pxd1-YFH(leu1) his3-D1 pxd1Δ::natMX cdt2Δ::kanMX spd1Δ::hphMX cdc25-22*	2
DY23099	h+	*leu1-32::pJK148-pxd1-YFH(leu1) his3-D1 pxd1Δ::natMX ddb1Δ::kanMX spd1Δ::hphMX cdc25-22*	2
DY7791	h+	*leu1-32 his3-D1 cdc25-22 pxd1-TAP::hphMX ddb1Δ::kanMX spd1Δ::hphMX*	2
DY7794	h+	*leu1-32 his3-D1 cdc25-22 pxd1-TAP::hphMX spd1Δ::hphMX*	2
DY7796	h+	*leu1-32 his3-D1 cdc25-22 pxd1-TAP::hphMX cdt2Δ::kanMX spd1Δ::hphMX*	2
DY22866	h+	*leu1-32::pJK148-pxd1-YFH(leu1) his3-D1 pxd1Δ::natMX spd1Δ::hphMX*	s2
DY22867	h+	*leu1-32::pJK148-pxd1-YFH(leu1) his3-D1 pxd1Δ::natMX cdt2Δ::kanMX spd1Δ::hphMX*	s2
DY22863	h+	*leu1-32::pJK148-pxd1-YFH(leu1) his3-D1 pxd1Δ::natMX ddb1Δ::kanMX spd1Δ::hphMX*	s2
DY4576	h+	*leu1-32 his3-D1 pxd1-TAP::hphMX*	s2
DY4759	h-	*leu1-32 his3-D1 pxd1-TAP::hphMX cdt2Δ::kanMX*	s2

DY19073	h+	*leu1-32 his3-D1? ars1::pREP1-6xHis-myc-Ubiquitin-natMX mts2-1 nda3-KM311*	2
DY19772	h-	*leu1-32::pDual-P41nmt1-pxd1-TAP(leu1+) his3-D1? mts2-1 nda3-KM311*	2
DY19081	h-	*leu1-32::pDual-P41nmt1-pxd1-TAP(leu1+) his3-D1? ars1::pREP1-6xHis-myc-Ubiquitin-natMX mts2-1 nda3-KM311*	2
DY19055	h-	*leu1-32::pDual-P41nmt1-pxd1-TAP(leu1+) his3-D1? ars1::pREP1-6xHis-myc-Ubiquitin-natMX mts2-1 nda3-KM311 spd1Δ::hphMX*	2
DY19053	h-	*leu1-32::pDual-P41nmt1-pxd1-TAP(leu1+) his3-D1? ars1::pREP1-6xHis-myc-Ubiquitin-natMX mts2-1 nda3-KM311 cdt2Δ::kanMX spd1Δ::hphMX*	2
DY15029	h+	*leu1-32::pJK148-pxd1-YFH(leu1)his3-D1 pxd1Δ::natMX pcn1-D122A::hphMX*	3
DY26551	h+	*leu1-32::pDual-P41nmt1-pxd1-GFP(leu1+) his3-D1 pxd1Δ::natMX*	3, S3
DY27735	h+	*leu1-32::pDual-P41nmt1-pxd1-(74-351)-GFP(leu1+) his3-D1 pxd1Δ::natMX*	S3
DY26543	h+	*leu1-32::pDual-P41nmt1-pxd1-(1-73)-NLS-GFP(leu1+) his3-D1 pxd1Δ::natMX*	S3
DY15987	h+	*leu1-32::pDual-P41nmt1-pxd1-(1-60)-NLS-GFP(leu1+) his3-D1 pxd1Δ::natMX*	S3
DY15998	h+	*leu1-32::pDual-P41nmt1-pxd1-(20-73)-NLS-GFP(leu1+) his3-D1 pxd1Δ::natMX*	S3
DY26540	h+	*leu1-32::pDual-P41nmt1-pxd1-(1-73)-NLS-GFP(leu1+) his3-D1*	S3
DY26542	h+	*leu1-32::pDual-P41nmt1-pxd1-(1-73)-NLS-GFP(leu1+) his3-D1 pxd1Δ::natMX*	S3
DY26537	h+	*leu1-32::pDual-P41nmt1-pxd1-(1-73)-NLS-GFP(leu1+) his3-D1 pcn1-D122A::hphMX*	S3
DY26538	h+	*leu1-32::pDual-P41nmt1-pxd1-(1-73)-NLS-GFP(leu1+) his3-D1 cdt2Δ::kanMX spd1Δ::hphMX*	S3
DY26544	h+	*leu1-32::pDual-P41nmt1-pxd1-(1-73)-PIP4A-NLS-GFP(leu1+) his3-D1*	S3

DY16291	h+	*leu1-32::pDual-P41nmt1-pxd1-(1-73)-K69A-NLS-GFP(leu1+) his3-D1*	S3
DY26287	h+	*leu1-32::pDual-P41nmt1-pxd1-(1-73)-PIP5A-NLS-GFP(leu1+) his3-D1*	S3
DY26281	h-	*leu1-32::pDual-P41nmt1-pxd1-GFP(leu1+) his3-D1*	3
DY26282	h-	*leu1-32::pDual-P41nmt1-pxd1-PIP4A-GFP(leu1+) his3-D1*	3
DY26358	h-	*leu1-32::pDual-P41nmt1-pxd1-PIP5A-GFP(leu1+) his3-D1*	3
DY19057	h-	*leu1-32::pdual-P81nmt1-pxd1-TAP (leu1+) his3-D1? ars1::pREP1-6xHis-myc-Ubiquitin-natMX mts2-1 nda3-KM311*	3
DY19754	h-	*leu1-32::pdual-P81nmt1-pxd1-TAP (leu1+) his3-D1? mts2-1 nda3-KM311*	3
DY19060	h-	*leu1-32::pdual-P81nmt1-pxd1-PIP5A-TAP (leu1+) his3-D1? ars1::pREP1-6xHis-myc-Ubiquitin-natMX mts2-1 nda3-KM311*	3
DY19065	h-	*leu1-32::pdual-P81nmt1-pxd1-PIP4A-TAP (leu1+) his3-D1? ars1::pREP1-6xHis-myc-Ubiquitin-natMX mts2-1 nda3-KM311*	3
DY17628	h+	*leu1-32::pdual-Propxd1-pxd1-PIP5A-GFP (leu1+) his3-D1 pxd1Δ::natMX*	4
DY17630	h+	*leu1-32::pdual-Propxd1-pxd1-PIP5A-Δ(108-226) -GFP (leu1+) his3-D1 pxd1Δ::natMX*	4, S4
DY17632	h+	*leu1-32::pdual-Propxd1-pxd1-PIP5A-Δ(302-348) -GFP (leu1+) his3-D1 pxd1Δ::natMX*	4
DY8233	h-	*leu1-32 his3-D1? dna2-C2*	S4
DY8236	h-	*leu1-32 his3-D1? dna2-C2 pxd1Δ::natMX*	4, S4
DY4325	h+	*leu1-32 his3-D1 cdc24-TAP::kanMX*	S4
DY6991	h-	*leu1-32 his3-D1 dna2-TAP::hphMX*	4, 5, S4
DY7124	h-	*leu1-32 his3-D1 dna2-TAP::hphMX pxd1Δ::natMX*	4, S4
DY7200	h-	*leu1-32 pfh1-R23 dna2-TAP::hphMX*	4
DY4597	h+	*leu1-32 his3-D1 pxd1Δ::natMX*	4

DY17612	h+	*leu1-32::pdual-Propxd1-pxd1-GFP (leu1+) his3-D1 pxd1Δ::natMX*	4, S4
DY17619	h+	*leu1-32::pdual-Propxd1-pxd1-M(108-226)D-GFP (leu1+) his3-D1 pxd1Δ::natMX*	4
DY17622	h+	*leu1-32::pdual-Propxd1-pxd1-Δ(302-348)-GFP (leu1+) his3-D1 pxd1Δ::natMX*	4
DY7550	h+	*leu1-32 his3-D1 cdt2Δ::kanMX pxd1Δ::natMX*	5
DY6248	h+	*leu1-32 his3-D1 ddb1Δ::kanMX pxd1Δ::natMX*	s5
DY7124	h-	*leu1-32 his3-D1 dna2-TAP::hphMX pxd1Δ::natMX*	5
DY22880	h+	*leu1-32::pdual-Propxd1-pxd1-Δ(302-348)-GFP (leu1+) his3-D1 cdt2Δ::kanMX pxd1Δ::natMX*	5
DY22876	h+	*leu1-32::pdual-Propxd1-pxd1-Δ(108-226) -GFP (leu1+) his3-D1 cdt2Δ::kanMX pxd1Δ::natMX*	S5
LD328	h+	*leu1-32 his3-D1*	5
DY4597	h+	*leu1-32 his3-D1 pxd1Δ::natMX*	5
DY7404	h+	*leu1-32 his3-D1 pxd1Δ::natMX spd1Δ::hphMX*	5
DY7413	h+	*leu1-32 his3-D1 cdt2Δ::kanMX*	5
DY7549	h+	*leu1-32 his3-D1 cdt2Δ::kanMX pxd1Δ::natMX*	5
DY7552	h+	*leu1-32 his3-D1 cdt2Δ::kanMX spd1Δ::hphMX*	5
DY22871	h+	*leu1-32::pdual-Propxd1-pxd1-GFP (leu1+) his3-D1 cdt2Δ::kanMX spd1Δ::hphMX pxd1Δ::natMX*	5
DY22875	h+	*leu1-32::pdual-Propxd1-pxd1-Δ(108-226)-GFP (leu1+) his3-D1 cdt2Δ::kanMX spd1Δ::hphMX pxd1Δ::natMX*	5
DY22878	h+	*leu1-32::pdual-Propxd1-pxd1-Δ(302-348)-GFP (leu1+) his3-D1 cdt2Δ::kanMX spd1Δ::hphMX pxd1Δ::natMX*	5
DY9453	h-	*leu1-32 his3-D1 psp3::kanMX,isp6::hphMX rad16-TAP::hphMX pxd1Δ::natMX*	6
DY20168	h+	*leu1-32::pdual-Propxd1-pxd1-Δ(302-348)-GFP (leu1+) his3-D1 psp3::kanMX,isp6::hphMX rad16-TAP::hphMX pxd1Δ::natMX*	6
DY20172	h+	*leu1-32::pdual-Propxd1-pxd1-Δ(302-348)-GFP (leu1+) his3-D1 psp3::kanMX,isp6::hphMX rad16-TAP::hphMX pxd1Δ::natMX cdt2Δ::kanMX spd1Δ::hphMX*	6

DY20173	h+	*leu1-32::pdual-Propxd1-pxd1-Δ(302-348)-GFP (leu1+) his3-D1 psp3::kanMX,isp6::hphMX rad16-TAP::hphMX pxd1Δ::natMX pcn1-D122A::hphMX*	6
DY20169	h+	*leu1-32::pdual-Propxd1-pxd1-PIP5A-Δ(302-348)-GFP (leu1+) his3-D1 psp3::kanMX,isp6::hphMX rad16-TAP::hphMX pxd1Δ::natMX*	6
DY26604	h+	*leu1-32 his3-D1 ade6-M210::pDual-Propxd1-pxd1-GFP(ade6+) urg1::Purg1lox-HO LEU-HOcs-His3-EU2-hsr1(his3+):: pDB3672 (natMX-svem) rad11(rpa1)-CFP::kanMX pxd1Δ::kanMX*	*6*
DY26592	h+	*leu1-32 his3-D1 ade6-M210::pDual-Propxd1-pxd1-PIP4A-Δ(302-348) -GFP(ade6+) urg1::Purg1lox-HO LEU-HOcs-His3-EU2-hsr1(his3+):: pDB3672 (natMX-svem) rad11(rpa1)-CFP::kanMX pxd1Δ::kanMX*	6
DY26596	h+	*leu1-32 his3-D1 ade6-M210::pDual-Propxd1-pxd1-PIP4A-Δ(108-226)-GFP (ade6+) urg1::Purg1lox-HO LEU-HOcs-His3-EU2-hsr1(his3+)::pDB3672(natMX-svem) rad11(rpa1)-CFP::kanMX pxd1Δ::kanMX*	6
DY26600	h+	*leu1-32 his3-D1 ade6-M210::pDual-Propxd1-pxd1-PIP4A-GFP(ade6+) urg1::Purg1lox-HO LEU-HOcs-His3-EU2-hsr1(his3+):: pDB3672 (natMX-svem) rad11(rpa1)-CFP::kanMX pxd1Δ::kanMX*	6
