# Supplementary material for: CRL4Cdt2 ubiquitin ligase regulates Dna2 and Rad16 (XPF) nucleases by targeting Pxd1 for degradation
Source: PLoS Genet. 2020 Jul 21;16(7):e1008933. doi: 10.1371/journal.pgen.1008933 (PMC7394458; doi:10.1371/journal.pgen.1008933)
Supplement: S2 Table — (PDF) [file pgen.1008933.s007.pdf]

S2 Table. Plasmids used in this study.

Plasmid	Description
pDB454	pJK148-Propxd1-Pxd1-YFH-leu1
pDB624	pAW8ENdeI-HO
pDB2341	pDual-Pnmt1-Pxd1-TAP
pDB2343	pDual-P41nmt1-Pxd1-TAP
pDB2669	pREP1-6xHis-myc-Ubiquitin-natMX
pDB3630	pDual-Propxd1-Pxd1-GFP
pDB3631	pDual- Propxd1-Pxd1-*Δ*(108-226)-GFP
pDB3633	pDual-Propxd1-Pxd1-*Δ*(302-348) -GFP
pDB3635	pDual-Propxd1-Pxd1-PIP5A-GFP
pDB3637	pDual-Propxd1-Pxd1-PIP5A-*Δ(*108-226)-GFP
pDB3639	pDual-Propxd1-Pxd1-PIP5A-*Δ*(302-348)-GFP
pDB3641	pDual-Propxd1-Pxd1-PIP5A-*Δ*(302-348)-GFP(ade6)
pDB3643	pDual-Propxd1-Pxd1-PIP5A-*Δ*(108-226)-GFP(ade6)
pDB3645	pDual-Propxd1-Pxd1-PIP5A-GFP(ade6)
pDB3647	pDual-Propxd1-Pxd1-GFP(ade6)
pDB3649	pDual-P41nmt1-Pxd1-(1-60)-NLS-GFP
pDB3651	pDual-P41nmt1-Pxd1-GFP
pDB3653	pDual-P41nmt1-Pxd1(20-73)-NLS-GFP
pDB3655	pDual-P41nmt1-Pxd1(1-73)-NLS-GFP
pDB3656	pDual-Pnmt1-Pxd1-PIP5A-TAP
pDB3658	pDual-Pnmt1-Pxd1-PIP4A-TAP
pDB3662	pBSK-Pcs2-LEU-His3-HO-EU2-Hsr1-5'UTR
pDB3671	pDual-Pnmt1-Pxd1-TAP
pDB3672	pFA6a-TAP-natMX(SVEM)-Hsr1-UTR-GC donor1-HpaI
pDB3678	pDual-P41nmt1-Pxd1(74-351)-GFP
