# Supplementary material for: CRL4Cdt2 ubiquitin ligase regulates Dna2 and Rad16 (XPF) nucleases by targeting Pxd1 for degradation
Source: PLoS Genet. 2020 Jul 21;16(7):e1008933. doi: 10.1371/journal.pgen.1008933 (PMC7394458; doi:10.1371/journal.pgen.1008933)
Supplement: Attachment — Submitted filename: Response_to_Reviewers_06062020.docx [file pgen.1008933.s008.docx]

Reviewer #1: The authors previously identified Pxd1 as an activator of Rad16 and an inhibitor of Dna2 nuclease activities. The current manuscript is a well executed study which is also well written. Results presented expand on the previous study and suggest that CRL4Cdt2 targets Pxd1 for degradation during S-phase, promoting Dna2 function and restricting Rad16 activity.

Suggestions for improvement:

1. Could the authors comment/speculate on the mechanism of Pxd1-dependent Dna2/Rad16 regulation? Have they looked at Dna2 and Rad16 levels throughout the cell cycle in their different genetic backgrounds to see if they are affected?

**Response: We thank the reviewer for the suggestions.**

**For the mechanisms of Pxd1-mediated regulation of Rad16 and Dna2, we showed in our previous PLOS Biology paper (PMID: 25203555) that Pxd1 regulates Rad16 and Dna2 by influencing their enzymatic activities, i.e., activating the 3′ endonuclease activity of Rad16 and inhibiting the RPA-mediated activation of the 5′ endonuclease activity of Dna2. Using an in vitro nuclease activity assay, we showed that recombinant Pxd1 purified from *E. coli* strongly stimulated the 3′ endonuclease activity of Rad16 in a manner dependent on the ability of Pxd1 to interact with Rad16 (Figure 5 in PMID: 25203555). We also showed that recombinant Pxd1 purified from *E. coli* prevented RPA from activating Dna2 by blocking the formation of the RPA-Dna2-DNA complex (Figure 6 in PMID: 25203555). More in-depth mechanistic details of how Pxd1 regulates nuclease activities will have to await future structural studies.**

**Regarding the protein levels of the nucleases, we showed in the previous paper that the complete loss of Pxd1 did not affect the protein levels of Rad16 and the Dna2 cofactor Cdc24 (Figure 1 in PMID: 25203555). In our current manuscript, we also showed that the level of Rad16 in HU-arrested S-phase cells remained the same regardless of whether Pxd1 was present or not (Figure 6). Thus, the presence/absence of Pxd1 does not appear to affect the protein levels of the nucleases regulated by Pxd1.**

2. It is difficult to analyse/review the results of the tetrad analyses. While the interpretation of the genotypes is shown in the figures, without knowing which phenotypes where scored for each colony and how, this can not be verified. Would it be possible to provide this info in the supplementary data?

**Response: In our laboratory, when the genotypes of tetrads are determined by replica plating on selective medium plates, we record the results in a tetrad scoring sheet without taking pictures of the replica plates. This seems to be a common practice as the two yeast laboratories where Li-Lin Du obtained his PhD and postdoctoral trainings (Peter Novick’s lab and Paul Russell’s lab, respectively) also record tetrad genotypes this way. For this reason, we cannot provide images of the replica plates for the reviewer to verify the genotypes. All strains used in this study will be available to other researchers, and it should be easy to reproduce our tetrad results by doing crosses.**

3. Fig. 3F: can relatively small reduction in PCNA/Pxd1 interaction in PIP4A and Pip5A account for lack of degradation?

**Response: We thank the reviewer for this question. This is a good point. We agree with the reviewer that the apparent weak PCNA-binding defects of the PIP degron mutants need some explanation.**

**Havens and Walter (PMID: 19595719) showed that degradation mediated by a PIP degron requires its interaction with DNA-bound PCNA, not free PCNA. The pull-down experiment shown in Fig. 3F cannot distinguish whether the observed Pxd1-PCNA interaction was happening with free PCNA or DNA-bound PCNA. It is possible that the residual PCNA binding by the Pxd1-PIP4A and Pxd1-PIP5A mutants is due to PIP degron-independent interactions between Pxd1 and free PCNA. In addition, the conditions of the pull-down experiment were not optimized for the detection of the binding with DNA-bound PCNA and thus might have underestimated the PIP degron-mediated interaction with DNA-bound PCNA. Furthermore, because the PIP degron and PCNA together constitute an interface for Cdt2 binding, it is possible that Pxd1-PIP4A and Pxd1-PIP5A mutations more severely hinder Cdt2 binding than PCNA binding. These possibilities are non-exclusive. We have now added these explanations in the revised manuscript.**

4. Fig. 3D: what do X and B symbols mean? There seem to be several AAs that do not conform to the consensus sequence.

**Response: X means any amino acid and B means K or R. We have added the meaning of the X symbol in the revised manuscript. Indeed, Pxd1(58-69) does not strictly conform to the consensus of the PIP degron. This lack of conformation to the consensus is common among known PIP degrons, and the sequences of the PIP degrons in the two previously known CRL4^Cdt2^ substrates in fission yeast, Cdt1 and Spd1, all deviate substantially from the consensus (please see Figure 2 in a review article by Havens and Walter, PMID: 21828267). We have now added a sentence in the revised manuscript to describe this common lack of conformation to the consensus.**

5. Fig. 6 a&b: Can the authors be sure that the observed nuclease activity is from Rad16 and not from a contaminating nuclease activity? Have the authors used a control (e.g. using untagged Rad16)?

**Response: We performed the in vitro nuclease assay using the exactly same protocol previously used in our PLOS Biology paper (PMID: 25203555). In that paper, we showed that the 3′ endonuclease activity is not due to contaminating nucleases by using an untagged control (Fig. 5A in PMID: 25203555).**

6. Could the authors discuss if they think the mechanism has been conserved (e.g. in human cells). Are there any Pxd1 homologues in (higher) eukaryotes?

**Response: In our previous PLOS Biology paper (PMID: 25203555), we proposed that Pxd1 may be a homolog of metazoan Slx4, as Pxd1 shares a weak sequence similarity with the XPF-binding region of metazoan Slx4 proteins. To our knowledge, there has been no report on the cell cycle regulation of the protein abundance of metazoan Slx4 proteins. We have added discussion on the possibility of evolutionary conservation in the revised manuscript.**

Reviewer #2: In a landmark 2014 paper published in PLoS Biology (reference 11), these authors reported the discovery of Pxd1, which they showed is a scaffolding protein that activates Rad16(XPF) and inhibits Dna2 structure specific endonucleases in fission yeast. Since then a key unanswered question has been why does Pxd1 exist? There is no apparent strategic purpose in constitutively activating Rad16 or inhibiting Dna2. The current study answers this question by showing that Pxd1 in specifically degraded in S-phase, thereby allowing cell cycle regulation of Rad16 and Dna2 activities. A thorough set of genetic and biochemical studies show that degradation of Pxd1 is mediated by CRL4/Cdt2 ubiquitin ligase, which was previously shown to regulate other important activities during S-phase. As predicted, this regulation requires PCNA binding to a PCNA-binding degron motif on Pxd1. In an important set of assays, the authors show that abrogation of Pxd1 regulation by CRL4/Cdt2 ubiquitin ligase increases genome instability, most notably increasing SSA repair of a DSB.

This is an excellent paper that provides key insights into the regulation of SSEs, which is a highly topical question in the fields of DNA repair and genome protection. The technical qualities of the experiments are excellent, with appropriate controls and statistical analyses (although see note below). There is ample evidence for all the key conclusions in the manuscript. The Introduction and Discussion sections are written to a high standard, with appropriate citations and interpretations of the data.

Minor issues to be addressed:

1. The Introduction should include a mention that Rad16 and XPF are used as shorthand for the Rad16-Swi10 and XPF-ERCC1 nuclease complexes, respectively.

**Response: We thank the reviewer for this suggestion. We have revised the manuscript according to the suggestion.**

2. It is unclear why p-values were not included in graphs such as those in Figures 1 and 2. They are hardly necessary but should be added if easily calculated.

**Response: The results shown in Figure 1 and 2 are representative data from independent experiments. Because the results are clear-cut and striking, we did not perform statistical analysis.**

Reviewer #3: This paper adds Pxl1 to the list of proteins that are related to S phase function and are degraded in S phase by ubiquitylation by CRL4Cdt2. Pxl1 was previously shown to inhibit Dna2 and stimulate Rad16. The data here show clearly that inactivation of the ubiquitin ligase stabilizes Pxl1 in S phase, and a reduction in Pxl1 ubiquitylation is shown. The PIP-degron in Pxl1 required for its ubiquitylation is tracked down, and mutation of the degron stabilizes the protein and prevents its interaction with PCNA. Proteolysis of Pxl1 results in a reduction of Rad16 nuclease activity, and strains with stabilized Pxl1 show an increase in single strand annealing in an assay that measures the SSA/gene conversion ratio in the repair of a DSB. However, stabilization of Pxl1 alone isn’t shown to have a replication phenotype in a wt background, and to demonstrate an effect on viability it was necessary to combine stabilized Pxl1 with defective alleles of Dna2, which makes the biological significance of the degradation less clear.

The paper is clearly presented and the data are convincing. One limitation is that it is assumed that Okazaki fragment processing is the key S phase function that is affected by stabilized Pxl1 when combined with defective Dna2. However, this is just inferred from genetic assays looking at synthetic lethality and S phase execution is not directly examined as such. Although increased sensitivity to camptothecin is shown, it isn’t clear whether this is due to a repair defect or an effect on S phase. It would be of value to demonstrate a replication phenotype with wt Dna2 using more sensitive assays, for instance by analysing S phase progression, fork rate or fork-stalling assays. Given that Dna2 is involved in several processes as well as Okazaki fragment processing, such as stalled-fork processing and replication of ‘difficult-to-replicate’ regions, it would be interesting to know which of these is affected by Pxl1 stabilization in an otherwise wt background.

**Response: We agree with the reviewer that the lack of a strong Dna2-related phenotype when S-phase degradation of Pxd1 is blocked is a weakness of our study. We also agree with the reviewer that the mild CPT sensitivity phenotype of the pxd1-PIP5A mutant may not necessarily be due to a defect in the replication-related function of Dna2. We have revised the text of the manuscript to state the uncertainty in the explanation of this phenotype.**

**It is indeed desirable to understand in more details how the S-phase functions of Dna2 may be affected by non-degradable Pxd1. We are grateful to the reviewer for the constructive suggestions on replication-related assays. However, given that *pxd1* degron mutants lack a detectable growth defect and their mild CPT sensitivity may not necessarily be replication-related, we feel that an obvious phenotype is not guaranteed to be revealed by replication assays. Furthermore, a substantial investment of time and efforts is needed to properly set up and carry out the replication assays, which we have not done before in our laboratory. For these reasons, we decide to not pursue these assays during the revision of this manuscript and leave them for future investigations.**

Minor point:

Some of the Pxl1-YFP images appear to show foci of Pxl1 (eg Fig 2A, cdt1D, 2B, cdt2D spd1D) – is this real and does this reflect accumulation of Pxl1 at sites of DNA damage?

**Response: Indeed, Pxd1 can form nuclear foci. Pxd1 was probably recruited to spontaneous DNA damage sites in cdt2 deletion mutant cells and cdt2 spd1 double deletion mutant cells through its interactions with Dna2 and Cdc24, which can form nuclear foci at sites of DSBs (PMID: 23628481).**
